# How much do tumor stage and treatment explain socioeconomic inequalities in breast cancer survival? Applying causal mediation analysis to population-based data

**DOI:** 10.1007/s10654-016-0155-5

**Published:** 2016-05-10

**Authors:** Ruoran Li, Rhian Daniel, Bernard Rachet

**Affiliations:** Cancer Research UK Cancer Survival Group, Department of Non-communicable Disease Epidemiology, Faculty of Epidemiology and Population Health, London School of Hygiene and Tropical Medicine, London, WC1E 7HT UK; Department of Medical Statistics, Faculty of Epidemiology and Population Health, London School of Hygiene and Tropical Medicine, London, UK; Centre for Statistical Methodology, London School of Hygiene and Tropical Medicine, London, UK

**Keywords:** Breast cancer, Survival, Socioeconomic inequalities, Causal mediation, Population-based data, Tumour stage, Surgical treatment

## Abstract

Substantial socioeconomic inequalities in breast cancer survival persist in England, possibly due to more advanced cancer at diagnosis and differential access to treatment. We aim to disentangle the contributions of differential stage at diagnosis and differential treatment to the socioeconomic inequalities in cancer survival. Information on 36,793 women diagnosed with breast cancer during 2000–2007 was routinely collected by an English population-based cancer registry. Deprivation was determined for each patient according to her area of residence at the time of diagnosis. A parametric implementation of the mediation formula using Monte Carlo simulation was used to estimate the proportion of the effect of deprivation on survival mediated by stage and by treatment. One-third (35 % [23–48 %]) of the higher mortality experienced by most deprived patients at 6 months after diagnosis, and one tenth (14 % [−3 to 31 %]) at 5 years, was mediated by adverse stage distribution. We initially found no evidence of mediation via differential surgical treatment. However, sensitivity analyses testing some of our study limitations showed in particular that up to thirty per cent of the higher mortality in most deprived patients could be mediated by differential surgical treatment. This study illustrates the importance of using causal inference methods with routine medical data and the need for testing key assumptions through sensitivity analyses. Our results suggest that, although effort for earlier diagnosis is important, this would reduce the cancer survival inequalities only by a third. Because of data limitations, role of differential surgical treatment may have been under-estimated.

**Electronic supplementary material:**

The online version of this article (doi:10.1007/s10654-016-0155-5) contains supplementary material, which is available to authorized users.

## Introduction

Substantial socioeconomic inequalities in cancer survival have been observed in England for decades [1–3], meaning that many cancer deaths could be avoided [4]. For breast cancer, besides lower screening uptake and differential access to treatment, more advanced stage at diagnosis and severe comorbidity are regularly proposed as the most plausible explanatory factors of these inequalities [5, 6]. However, both factors seem to explain only part of these inequalities, at least for breast and colorectum cancers [7, 8].

Population-based data are crucial to understand the mechanisms affecting all patients and to help define policies. Quantifying the proportion of the effect of deprivation on survival that’s attributable to differential stage of diagnosis and treatment is important for better resource allocation to address the gap between the rich and the poor. Methodological issues, however, are inherent to observational data. Most of the previous results were based on conventional analytic approaches (e.g. by describing the deprivation gap after adjusting for or stratifying by stage). However, if stage and treatment are on the causal pathway from deprivation to cancer survival, or if there is an interaction between deprivation and the mediator(s), these conventional approaches may lead to flaws in interpretation [9–12]. Using methods from the causal inference literature, we aim to disentangle the contributions of differential stage at diagnosis and differential treatment to the socioeconomic inequalities in cancer survival. To this end, we use population-based and routinely collected data for all patients diagnosed with a breast cancer within a defined area.

## Materials and methods

### Data

We included in the analyses all women (aged 15–99 at diagnosis) diagnosed with malignant, invasive breast cancer during 2000–2007, followed up until 31 December 2007, and collected by the Northern and Yorkshire Cancer Registry Information Service (NYCRIS), a population-based cancer registry covering 12 % of the English population. Ascertainment of the vital status was considered to be complete for all patients [13].

Each patient was allocated a socio-economic deprivation score according to her area (Lower Super Output Area) of residence at the time of diagnosis, using the English Indices of Multiple Deprivation (IMD) 2001 (income domain) [14]. These scores were categorised according to the quintiles of their national distribution.

Each patient was allocated one of the four broad tumor TNM stages using a restrictive approach [15].

Information on surgical treatment was retrieved from a routinely collected national hospital dataset (Hospital Episode Statistics or HES). We retained surgical treatment within 1 month before and 6 months after the cancer diagnosis. The treatment (OPCS-4) codes [16] were categorized based on recommendations made by the Site-Specific Clinical Reference Group (SSCRG) for breast cancer [17] (Appendix 1). These categories were then dichotomized into ‘major treatment’ (axillary dissection or other axillary nodal procedures, breast conserving surgery, mastectomy, and plastic surgery) and ‘minor or no surgery’ (other surgical procedures and none).

### Analyses

We estimated net survival from breast cancer, for each deprivation group and by stage, using the Pohar-Perme estimator [18] implemented in the Stata [19] package *stns* [20].

**Fig. 1:**
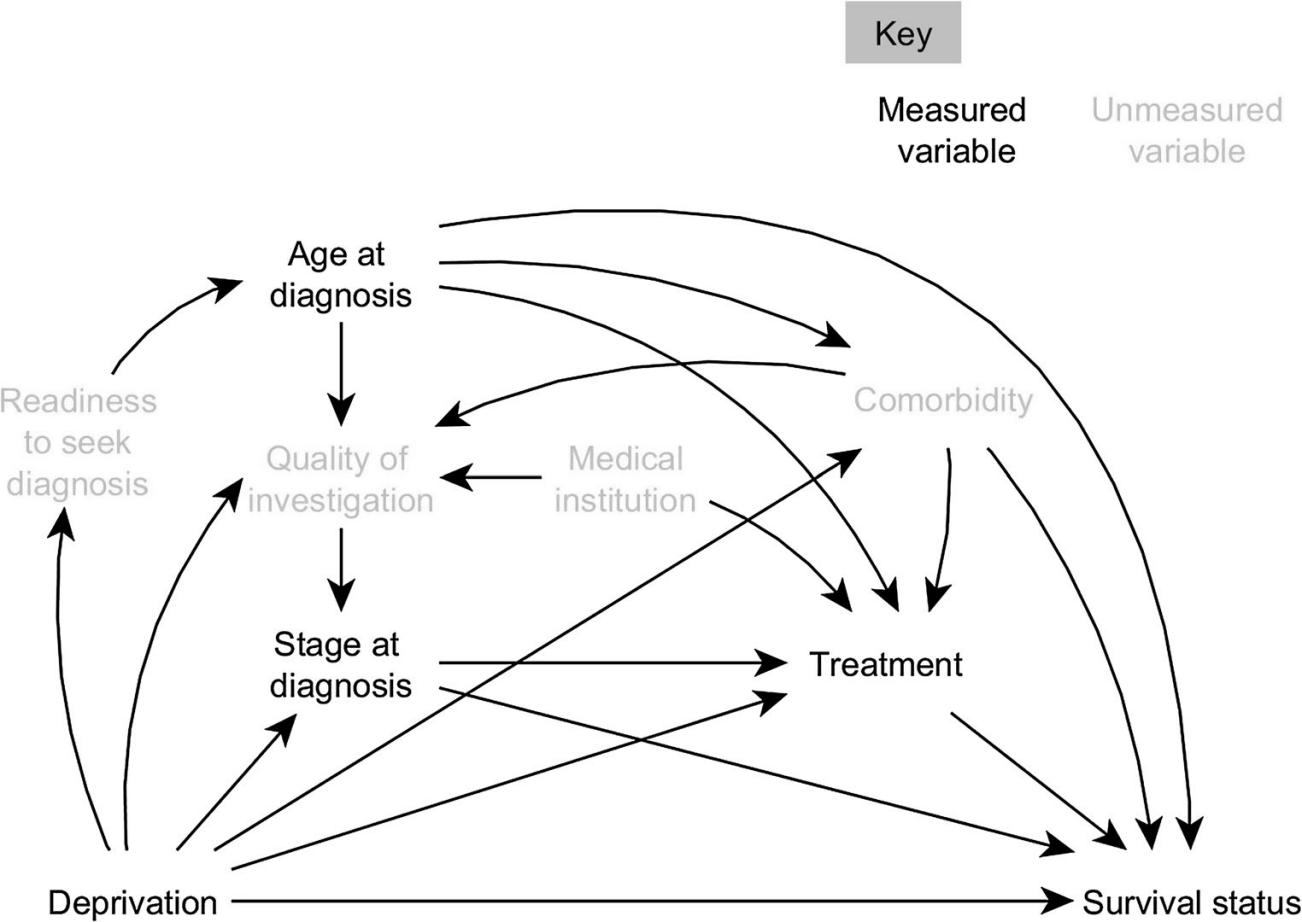
Direct Acyclic Diagram (DAG) depicting the causal relationships between deprivation and survival status in breast cancer patients. Year of diagnosis and region are considered as baseline confounders, with potentially an arrow to each node in the diagram, and thus are not shown in this DAG

The assumed causal relationships between variables are represented by a Directed Acyclic Graph (DAG) (Fig. 1, Appendix 2). Our main exposure of interest, the patient’s deprivation level, causally influences the age at which a woman was diagnosed with breast cancer, her comorbidity, thoroughness of the disease investigation, stage at diagnosis, the treatment received, and survival status after the cancer diagnosis. Year and regions at diagnosis were considered as baseline confounders. Factors such as the quality of investigation and comorbidity (shown in grey as unmeasured variables) were incorporated in the DAG. The omission of variables and arrows also represents our causal assumptions, e.g. we assume that the quality of the investigation does not affect survival except through its effect on stage at diagnosis.

We examined what proportions of the deprivation gap in survival were explained separately by tumor stage and treatment. Because of our data structure (in particular, the existence of important mediator-outcome confounders affected by exposure, the likely presence of many interactions and the fact that our outcome is binary) we focused on the decomposition of the *total causal effect* (TCE) into what have recently been termed *randomized interventional analogues of natural direct and indirect effects*, henceforth RIANDE and RIANIE [21–23].

The RIANDE and RIANIE can be estimated with an extension of Robins’ g-computation formula [24] implemented using Monte Carlo simulation in the Stata command *gformula* [25]. We chose this method because of flexible modelling that allows interactions and other non-linearities. Although flexible in terms of parametric modelling assumptions, this method relies on the assumptions of no unaccounted confounding of the exposure–mediator, mediator–outcome or exposure–outcome relationship.

We conducted three analyses to investigate the mediating roles of stage and treatment (Appendix 3, Appendix 4). We first estimated the proportion of the effects of deprivation on survival that was mediated by differences in stage at diagnosis, i.e. we computed the ratio between the effect of deprivation on log odds of death that was mediated by stage (the *RIANIE*) and the total effect of deprivation on log odds of death (the *total causal effect, TCE*, which is the sum of the *RIANDE*—the effect not mediated by the mediator stage—and the *RIANIE*). In the second analysis, we estimated the proportion of the effect of deprivation on log odds of death that was mediated by differences in treatment. Stage at diagnosis was here considered to be a confounder of the relationship between treatment and survival, and was allowed to be affected by deprivation. Such a confounder is dealt with using an extension of the g-computation formula [24, 25]. In the third analysis, we estimated the proportion of the effect of deprivation on treatment that is mediated by differential stage.

Because the deprivation gap in survival varies by time since diagnosis, the binary survival outcome (dead vs. alive) was stratified according to time since diagnosis: at 6 months, 1 year given (conditioning on) 6-month survival, 3 years given 1-year survival, and 5 years given 3-year survival. The analyses were performed separately on each of these four binary survival outcomes, in order to disentangle early from late mediating effects of stage and treatment on deprivation gap in survival.

We used multinomial regressions to model stage at diagnosis (four categories) and logistic regression for treatment and survival status. Age at diagnosis was modelled using restricted cubic splines [26].

Single stochastic imputation within the g-computation procedures was used to handle missing stage (8 %). All variables in the models (including vital status), exact length of follow-up times and detailed treatment categories were included in the imputation model.

## Results

We analyzed 36,793 women diagnosed with breast cancer between 2000 and 2007 in Yorkshire and North East (Table 1). More deprived patients were diagnosed at an older age (*P* = 0.001) and a more advanced stage (*P* < 0.001) than the more affluent. The higher the deprivation level, the more advanced the stage.

**Table 1:** Characteristics of women diagnosed with breast cancer, Yorkshire and North East (England), 2000–2007

	All patients	Deprivation				
		Least deprived	2	3	4	Most deprived
Number of patients	36,793	6411	7549	6982	7642	8209
% Alive at end of follow-up	78.9	85.1	81.2	79.2	76.7	73.6
Mean age at diagnosis	62.9	61.0	61.3	62.4	63.1	63.7
(interquartile range)	51.9–73.9	51.2–70.3	51.8–72.7	52.1–74.3	52.1–75.7	52.2–75.2
Stage at diagnosis (%)						
I	37.6	40.9	38.8	38.0	36.3	34.8
II	43.0	43.7	43.9	41.7	42.5	43.1
III	6.8	6.2	6.2	7.0	7.0	7.4
IV	4.6	3.5	3.7	4.6	5.0	5.9
Missing	8.0	5.7	7.4	8.7	9.2	8.8
% Receiving major treatment	26.9	25.9	25.7	27.6	27.1	28.0

### Survival from breast cancer

**Fig. 2:**
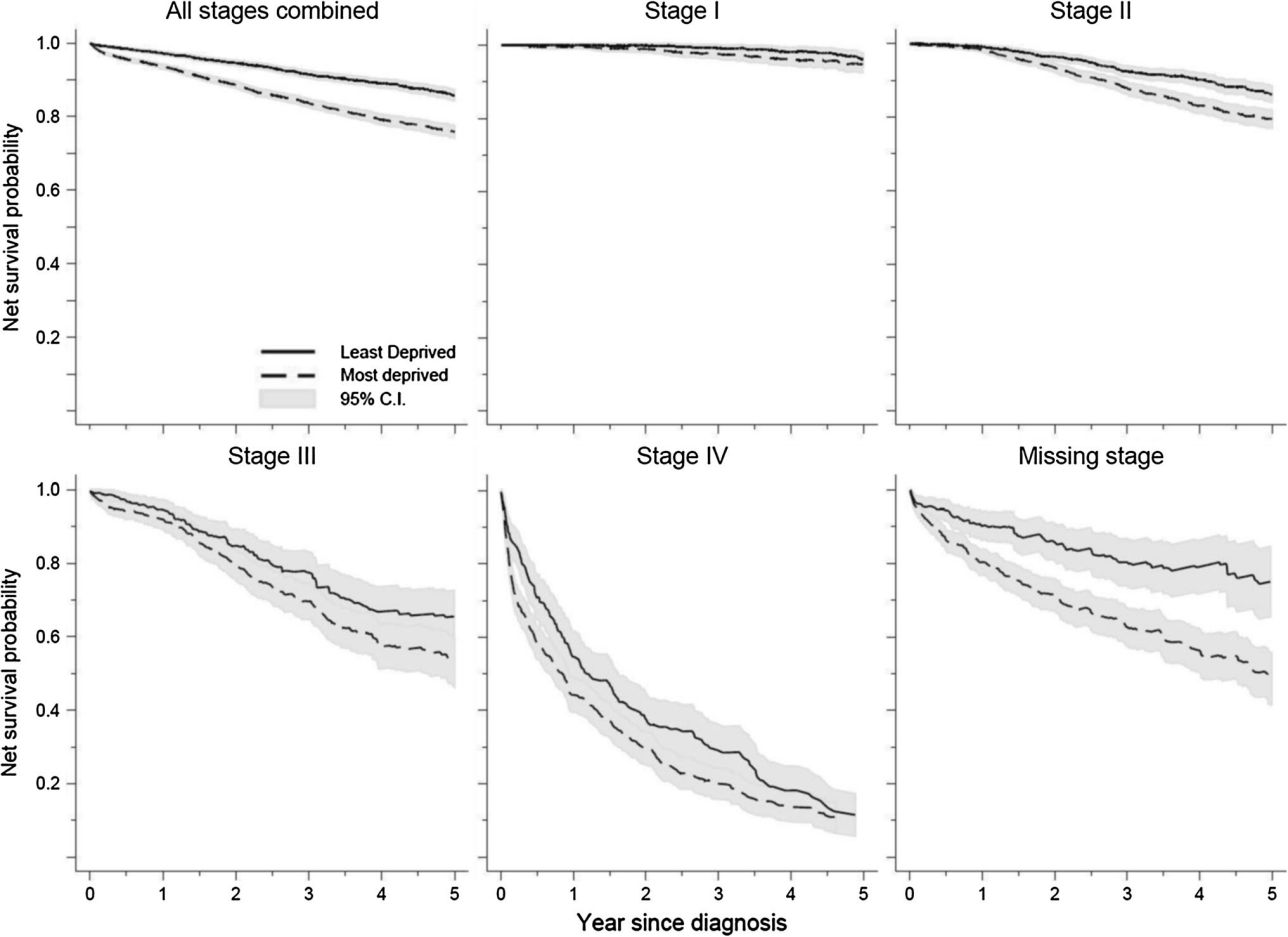
Net survival by deprivation and stage at diagnosis, women diagnosed with breast cancer, Yorkshire and North East (England), 2000–2007

Net survival differed between the most affluent and the most deprived patients by 3 % at 1 year (97 vs. 94 %), and 10 % at 5 years (86 vs. 76 %) after diagnosis (Fig. 2, Appendix 5).

Stage-specific survival estimates were consistently lower in the more deprived patients. Large deprivation gap existed for the short-term survival (at 1 year after diagnosis) in the most advanced stage (IV), and in the long-term survival (at 5 years) in the less advanced stages (II–III). For patients with missing stage information, the more deprived experienced worse survival.

### Total effect of deprivation on cancer survival status

**Fig. 3:**
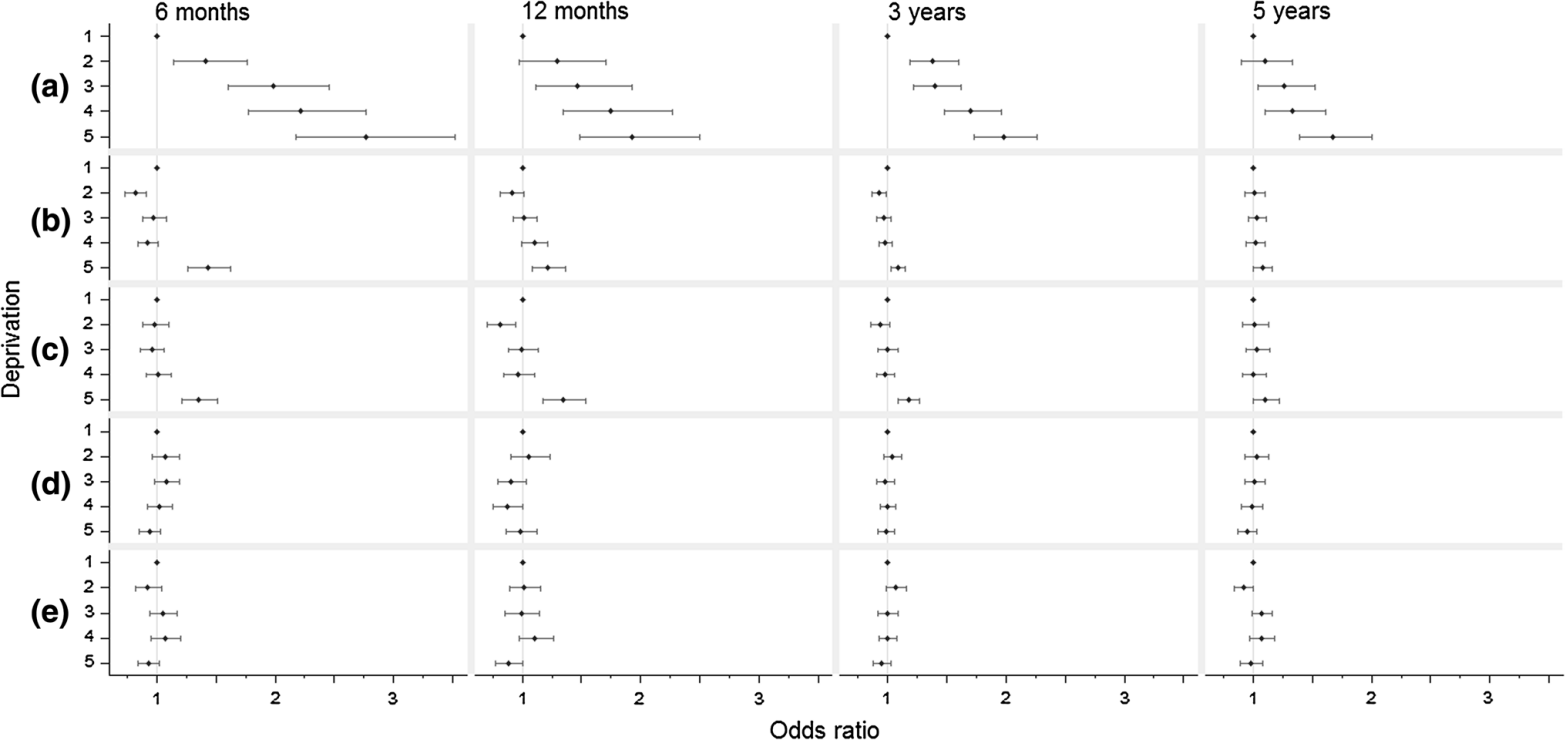
Effect of deprivation on odds of deaths at different times since breast cancer diagnosis, results from the G-computation Analyses. (*a*) Shows the total effect of increasing deprivation on odds of death. (*b*) shows the effect that were mediated via stage at diagnosis, and (*c*) shows the effect mediated via surgical treatment. Odds of death at 12 months since diagnosis are conditional on surviving the first 6 months after cancer diagnosis. Similarly, odds of death at 3 years are conditional on survival the first 12 months; odds of death at 5 years are conditional on survival the first 3 years. We used conditional odds of death in order to disentangle early from late mediating effects of stage and treatment on deprivation gap in survival

We first estimated the *total causal effect* of deprivation on survival status, which is the sum of all effects shown in Fig. 1, adjusted for the confounding effect of region and year of diagnosis. The odds of dying within the first 6 months since diagnosis roughly increased linearly with increasing deprivation (odds ratio [OR] comparing most deprived to most affluent patients: 2.77, 95 % confidence interval [CI] 2.17, 3.53) (Fig. 3a, Appendix 6). This deprivation effect decreased slightly as follow-up time increased. However, the effect remained fairly strong at 5 years conditioning on 3-year survival (for most deprived compared with least deprived, OR: 1.67, 95 % CI 1.39, 2.00).

### Role of stage on the socio-economic differences in cancer survival status

The effect of socioeconomic status on survival mediated through stage (the *RIANIE*, Fig. 3b) was only apparent when comparing the most deprived with the least deprived. This indirect effect through stage decreased as time since diagnosis increased (OR for 6-month mortality: 1.43, CI: 1.27, 1.62; OR for 5-year conditional mortality: 1.08, CI 1.00, 1.16) (Fig. 3b). On the log odds scale, stage only accounted for about one-third of the total effect of deprivation at 6-month and 1 year (proportion mediated [PM]: 35 %, CI 23, 48 %; 30 %, CI 5, 54 %, respectively), a proportion which decreased to just over a tenth at 3 and 5 years since diagnosis (PM: 12 %, CI 4, 21 %; 14 %, CI −3, 31 %, respectively) (Appendix 6).

We also treated both age and stage as mediators (in place of just stage). We assumed here linear associations between the logarithm of age and treatment or mortality. The overall pattern hardly changed although adding age tended to slightly increase the long-term PM. This might reflect the long-term effect of age on all-cause mortality (Appendix 6).

### Role of treatment on the socio-economic differences in cancer survival status

**Fig. 4:**
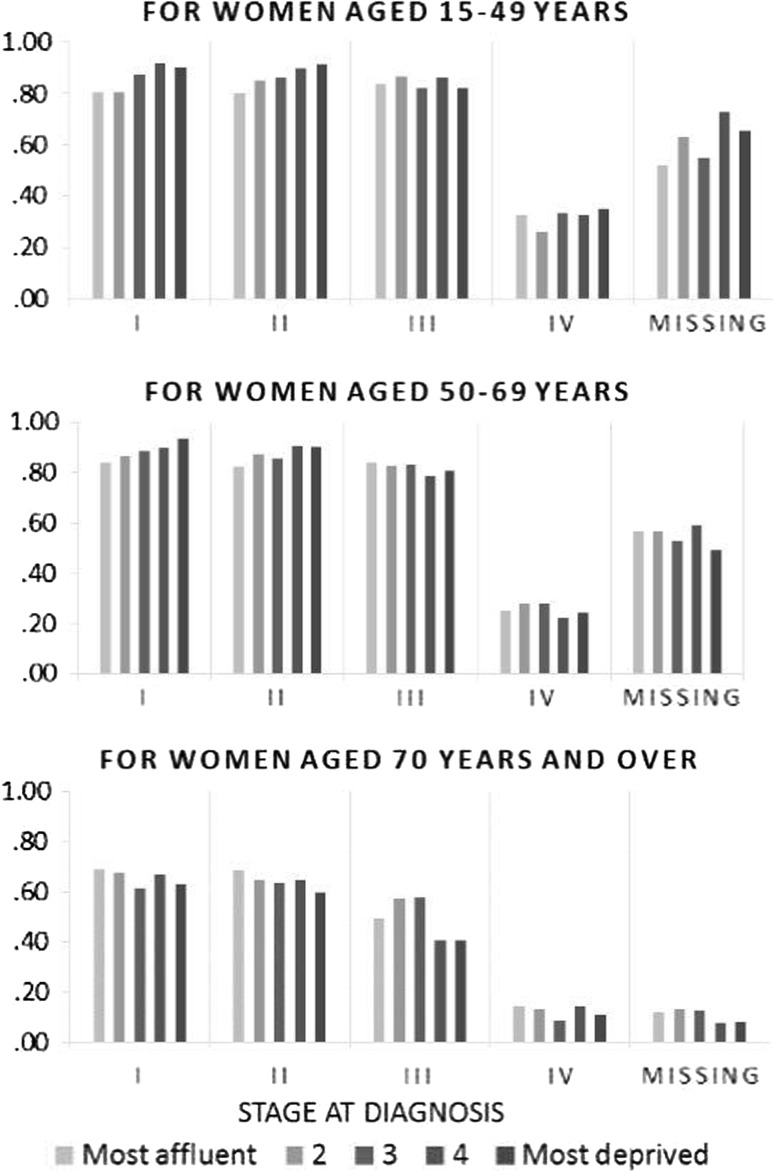
Probability of receiving major surgery for women diagnosed with breast cancer, Yorkshire and North East (England), 2000–2007

The higher the stage, the less likely a patient would receive major surgical treatment (Fig. 4). For patients under 70 years when diagnosed with early stages (stages I and II), more deprived patients received more treatment. By contrast, for patients aged 70 and over, more deprived patients received less treatment for all stages.

We did not find any evidence for total effect of deprivation and effect mediated by stage on treatment (Appendix 8). Although treatment patterns differ between different socioeconomic classes, the main mediation analysis found no evidence for the effect of deprivation on mortality mediated through differential treatment (Fig. 3c, Appendix 7).

## Discussion

Our results suggest that, for the most deprived patients only, earlier breast cancer diagnosis would reduce the deficit in short-term survival by up to a third and in longer-term survival by up to a tenth. The available crude information on treatment seems to show that differential surgical treatment between deprivation groups played a minor role in socioeconomic inequalities in breast cancer survival.

For the younger (15–69) patients diagnosed at stage I or II, the finding that more deprived patients received more treatment contradicts the *a prior* hypothesis by some oncologists: more deprived patients may have more comorbidity, and thus less aggressive diagnostic investigation and treatment. Prevalence of both obesity and tobacco smoking widely varies in the general population between deprivation groups [27, 28], but we did not have reliable information about comorbidity of the cancer patients. However, the surgical differences observed between the socioeconomic groups may reflect that, within a given stage, more deprived patients were diagnosed with more advanced disease. To investigate this hypothesis, we will need more detailed information on tumor stage and diagnostic investigation. In addition, more affluent patients may have received treatment within private facilities, information not available to us.

In the absence of individual measure of socioeconomic status for population-based studies in England, we used an ecological measure of deprivation [14]. Because LSOAs (the geographical level of the deprivation measure) are relatively small (1500 inhabitants on average) and have been made as socially homogenous as possible, the ecological bias is probably small. An ecological measure reflects both the individual and contextual dimensions of deprivation. We are not able to disentangle individual and contextual dimensions of deprivation and this affects conceptualizing hypothesized interventions. The English healthcare system is strongly territorialised, and any perceived intervention should primarily target these territories in which individual-level actions (via the general practices) are also possible. Such interventions correspond to our conceptual framework, i.e. we asked: what would be the outcome of women in the deprived group, had they lived in the same area as those lived in the most affluent areas, with similar background risk factors and access to primary and secondary healthcare for their cancer diagnosis and treatment.

We identified three main plausible reasons that could bias our results: misclassification of the stage at diagnosis, misclassification of the treatment and unmeasured confounders between the mediator(s) and the outcome(s).

### Misclassification of stage at diagnosis

More deprived cancer patients may more likely be managed by non-specialized centres and low-workload surgeons [29]. Evaluating the spread of their cancers (i.e. staging) may not be thorough enough (Fig. 1) and, as a result, they might be more often under-staged and receive non-optimal treatment [8]. We tested this hypothesis by assuming different proportions of the most deprived patients were under-staged. We randomly up-staged 10, 30 and 50 % of the most deprived patients by one level (stage I to II, etc.) ten times and reran the analyses to estimate the PM distributions. The proportion of survival inequalities mediated by stage hardly changed with 10 % of under-staged most deprived patients, but increased substantially with 30 and 50 % of under-staged, more particularly for conditional survival at 1 year and over (data on request). For example, more than half of the lower conditional 1-year survival among the most deprived patients would be mediated by stage if above 30 % of them were under-staged (vs. 30 % mediated if stage was not misclassified). Changing our main conclusion about the role of stage on survival inequalities would require above 30 % of the most deprived patients were systematically under-staged, compared to none in the most affluent group, a rather extreme assumption that is not supported by the literature.

### Misclassification of treatment

**Fig. 5:**
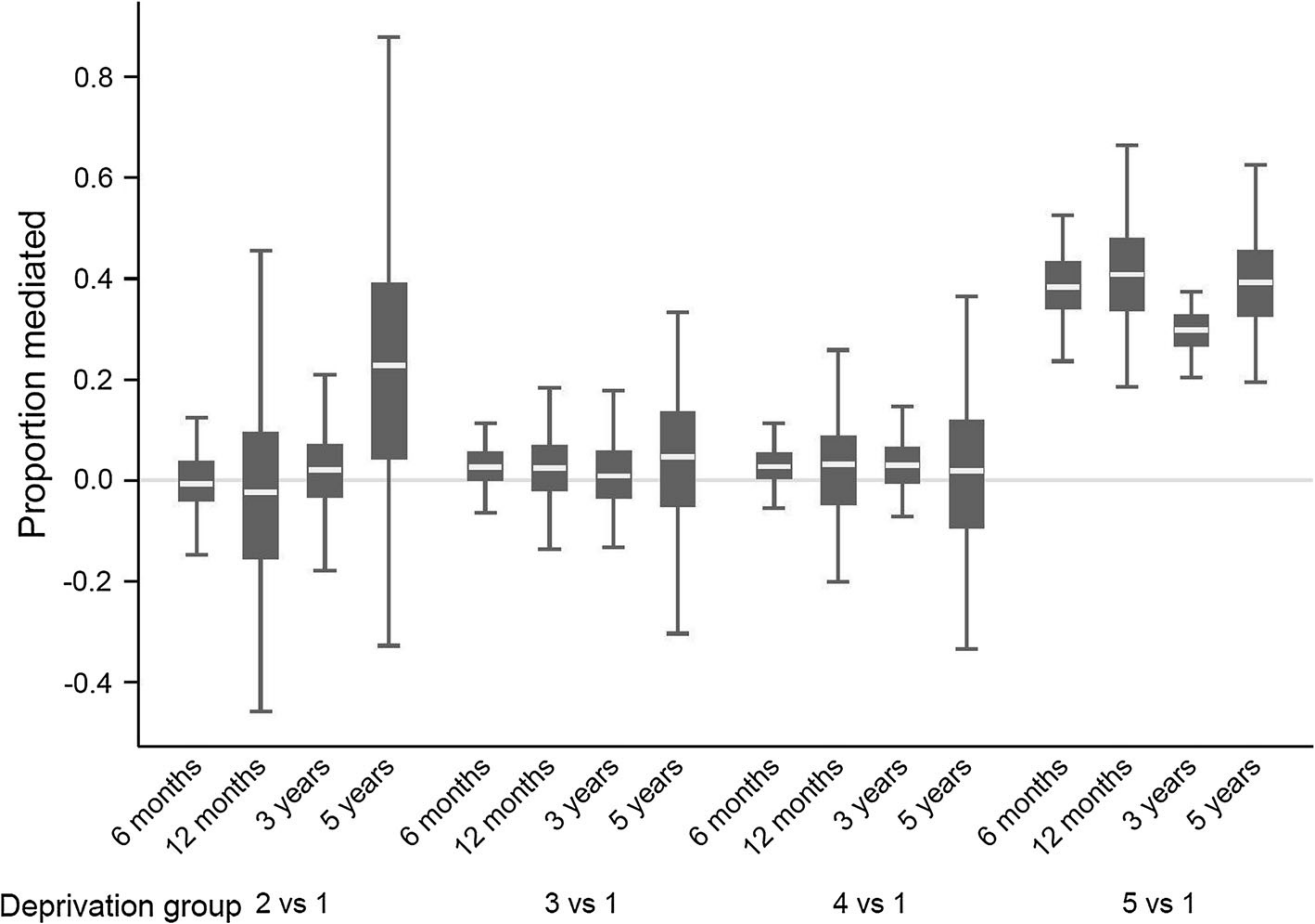
Proportion of the effect of deprivation on odds of death mediated by treatment in a sensitivity analysis taking into account of the potentially missing surgical information due to treatment in private hospitals

Surgery, often in conjunction with other treatments, remains the main curative treatment of breast cancer. Information on radiotherapy and chemo/hormono-therapy was too poor to be used here. The quality, completeness and intention (whether curative or not) of the surgical procedure were not known. It was reported that 3.6 % of surgical treatment for breast cancer were made in private hospital in NYCRIS [30]. Such under-estimation of the surgery proportion is likely to affect primarily the more affluent patients. We conducted a sensitivity analysis to investigate how such misclassification would influence the mediating effect of treatment on the socioeconomic differences in breast cancer survival. We randomly changed the treatment status for 3.6 % of the patients from no/minor surgical procedures to major surgery, according to the stage and age distribution of those who had records of receiving major treatment. We assumed that those patients were entirely from the most affluent group. We generated 100 new datasets on which we carried out g-computation analyses, estimating the proportion of effect of deprivation on survival mediated by treatment. We confirmed the absence of indirect effect through differential treatment on cancer survival status for deprivation groups 2–4. However, treatment did mediate around 30–40 % of the differential mortality between the most deprived patients and the most affluent, regardless time since diagnosis (Fig. 5), under the assumption that only the most affluent patients had surgeries in private hospitals. In addition, around 10 % of the cancer registry cases could not be matched with HES (inpatient data from the National Health Services) [30]. Surgical information is likely to be missing completely at random for such patients and we do not expect this to bias our results.

Our analyses crudely dichotomized treatment into ‘major’ and ‘minor or no’ surgery categories. When ‘major’ surgery was further categorized into ‘breast conserving surgery’ and ‘mastectomy’, the results remained unchanged (Appendix 7). Nevertheless, the crude treatment information may explain why the mediating effect of treatment on deprivation gap in survival remained moderate and was not affected in the sensitivity analysis on the misclassification of tumor stage.

### Unmeasured confounders

We assumed that, conditional on deprivation, age, stage and year at diagnosis, and government regions, there were no further (unmeasured) common causes of treatment and survival status. However, in addition to staging thoroughness, comorbidity could be an important confounder for treatment and mortality, which we did not account for due to lack of reliable individual information. Ignoring the confounding effect of comorbidity would potentially lead to over-estimation of the beneficial effect of major surgery on mortality: patients with high levels of comorbidity experience high mortality, and may have lower rate of major surgery. Since we found little evidence for treatment to mediate the effect between deprivation and mortality in the original dataset, inclusion of comorbidity would not change this overall interpretation, but only if stage and treatment were not misclassified. If reliable information on comorbidity becomes available, we could potentially treat it as a mediator between deprivation and mortality, and estimate how much contribution it has to the deprivation gap in survival.

## Concluding remarks

Our results are based on population-based data, i.e. on virtually all patients diagnosed with a breast cancer in a given region, including those who were diagnosed with advanced stage and those who were not optimally managed. Since our main focus is to better understand the causal relationships between deprivation and breast cancer survival, and to divide it into path-specific components, applying methods from the growing literature on causal mediation is highly appropriate.

To our knowledge, very few studies attempted to disentangle the effects of deprivation on breast cancer survival. Two studies used data from an earlier periods (late 1990s) of the same region as our study [31, 32]. A complete-case analysis found adverse stage distribution and less surgical treatment (even after adjustment for stage) among more deprived patients [31]. No stage-specific results were provided on treatment. Lower overall 5-year survival was associated with deprivation after adjustment for age and stage, but underlying pathways could not be deduced from these results. A second analysis using latent class modelling [32] clearly identified two groups of patients according to their prognosis: more advanced stage seemed to play a role in the deprivation gap in 5-year survival only in one group. The conclusions were weakened by the fact that overall survival was analysed, while mortality from causes other than breast cancer varies greatly by deprivation within 5 years since diagnosis. Our study is also based on overall mortality. Not adjusting for competing risks of death will dilute the mediating effect of stage. However, this effect would be minimal for short-term survival, as mortality from causes not related to breast cancer does not play a significant role in short-term survival status, especially at 6 months after diagnosis. Using conditional survival also reduced this bias.

Contrasting with our results, a study in another English region found that, in 2006–2010, adverse stage distribution explained half of the deficit in 5-year breast cancer relative survival observed among the most deprived patients, but all of it in other deprivation groups [33]. However, stage-standardisation, used in order to eliminate differences in stage distribution by deprivation, cannot fully identify the effect of deprivation mediated by tumor stage on such observational data.

Applying another causal inference approach, Valeri et al. [34] found that the contribution of stage to the disparities in survival from colorectal cancer between Blacks and Whites in the US was similar to our results for the socio-economic disparities in breast cancer survival in England. They however concluded that the mediation effect of stage represented a “substantial reduction” while we talked about a small reduction, which reflects differences in the study context. Contrasting with the US (at least until recently), the healthcare system in England is universal with free access to diagnosis and treatment. In theory, most disparities in cancer survival should be therefore due to patient and tumour factors, more specifically tumour stage at diagnosis and comorbidity, and not to healthcare system factors. Contrasting this belief, our results add to the growing evidence that one of the strongest prognostic factors, stage, plays a relatively small role in the socio-economic inequalities in cancer survival. Comorbidity (or health performance status) is likely to contribute to inequalities, but will reduce the stage contribution estimated further. It means that, in the context of a supposedly equitable healthcare system, a large proportion of these inequalities remain unexplained; inequalities within the healthcare system are likely to play a key role.

Despite data limitations, we were able to estimate the proportions of the deprivation gap in cancer survival mediated via tumor stage and treatment separately. It informs us about their respective roles, and ultimately, what may be done to most effectively reduce the deprivation gap in cancer survival. In particular, effort for earlier diagnosis would reduce the cancer survival inequalities only by a third. Our conclusions may, however, be altered by unmeasured confounders such as comorbidity, staging thoroughness and detailed treatment information, of which quality and completeness are improving dramatically in the population-based cancer registry data in England. The changes in results after sensitivity analyses demonstrate the vital importance of using reliable and correctly classified surgical treatment data in similar studies.

## Electronic supplementary material

Below is the link to the electronic supplementary material.

**Appendix 1.** Classification of Surgical Procedures. (PDF 216 kb)

**Appendix 2**. Potential Collider Bias by Studying Incident Breast Cancer Cases. (PDF 215 kb)

**Appendix 3.** Mathematical Explanations for the Three Mediation Analyses. (PDF 551 kb)

**Appendix 4.** Model specifications and STATA codes (PDF 431 kb)

**Appendix 5.** One-year and Five-year Net Survival for Women Diagnosed with Breast Cancer, Yorkshire and North East (England), 2000-07. (PDF 237 kb)

**Appendix 6:** Effects of Deprivation on Mortality, Mediated via Stage at Diagnosis. (PDF 325 kb)

**Appendix 7:** Effects of Deprivation on Mortality, Mediated via Surgical Treatment. (PDF 324 kb)

**Appendix 8:** Effects of Deprivation on Receiving Major Surgical Treatment, Mediated via Stage at Diagnosis. (PDF 308 kb)
