# Supplementary material for: How much do tumor stage and treatment explain socioeconomic inequalities in breast cancer survival? Applying causal mediation analysis to population-based data
Source: Eur J Epidemiol. 2016 May 10;31:603–11. doi: 10.1007/s10654-016-0155-5 (PMC4956701; doi:10.1007/s10654-016-0155-5)
Supplement: Supplementary file 1 — Appendix 1. Classification of Surgical Procedures. (PDF 216 kb) [file 10654_2016_155_MOESM1_ESM.pdf]

Appendix 1. Classification of surgical procedures

Where the are blanks, this procedure was classified as 'non-major' (or 'minor'). Non-major procedures were diagnostic, exploratory or procedures unrelated to the breast. A patient is classified as having "no surgery" if there's no OPCS-4 code.

Surgical type	Category	OPCS-4 codes	Note
Major	Axillary Dissection	T852	Block dissection of axillary lymph nodes
Major	Axillary Dissection	T858	Other specified block dissection of lymph nodes
Major	Axillary Dissection	T859	Unspecified block dissection of lymph nodes
Major	Breast Conserving Surgery	B281	Quadrantectomy of breast
Major	Breast Conserving Surgery	B282	Partial excision of breast, Partial mastectomy, WLE, includes wedge or segmental excision of breast NEC.
Major	Breast Conserving Surgery	B283	Excision of lesion of breast, includes lumpectomy, excision biopsy.
Major	Breast Conserving Surgery	B284	Re-excision of breast margins
Major	Breast Conserving Surgery	B285	Wire guided partial excision of breast
Major	Breast Conserving Surgery	B286	Excision of accessory breast tissue
Major	Breast Conserving Surgery	B288	Other specified other excision of breast
Major	Breast Conserving Surgery	B289	Unspecified other excision of breast
Major	Breast Conserving Surgery	B323	Wire-guided biopsy of lesion of breast
Major	Breast Conserving Surgery	B328	Localisation biopsy/excision.
Major	Breast Conserving Surgery	B341	Subareolar excision of mammillary duct
Major	Breast Conserving Surgery	B342	Excision of mammillary duct NEC
Major	Breast Conserving Surgery	B343	Excision of lesion of mammillary duct nec. Microdochectomy.
Major	Breast Conserving Surgery	B344	Microdochotomy
Major	Breast Conserving Surgery	B352	Excision of nipple
Major	Breast Conserving Surgery	B353	Extirpation/removal of lesion of nipple.
Major	Breast Conserving Surgery	B374	Capsulectomy of breast
Major	Breast Conserving Surgery	B378	Other specified other operations on breast
Major	Breast Conserving Surgery	B401	Interstitial laser destruction of lesion of breast
Major	Breast Conserving Surgery	B408	Destruction of lesion of breast, Other specified
Major	Breast Conserving Surgery	B409	Destruction of lesion of breast, Unspecified
Major	Other Axillary Nodal Procedure	O142	Sentinel lymph node
Major	Other Axillary Nodal Procedure	T862	Sampling of axillary lymph nodes
Major	Other Axillary Nodal Procedure	T868	Other specified sampling of lymph nodes
Major	Other Axillary Nodal Procedure	T869	Unspecified sampling of lymph nodes
Major	Other Axillary Nodal Procedure	T873	Excision or biopsy of axillary or supraclavicular nodes lymph nodes.
Major	Other Axillary Nodal Procedure	T878	Other specified excision or biopsy of lymph node
Major	Other Axillary Nodal Procedure	T879	Unspecified excision or biopsy of lymph node
Major	Other Axillary Nodal Procedure	T888	Other specified drainage of lesion of lymph node
Major	Other Axillary Nodal Procedure	T911	Biopsy of sentinel lymph node NEC
Major	Other Axillary Nodal Procedure	T912	Scanning of sentinel lymph node
Major	Other Axillary Nodal Procedure	T918	Other specified operations on sentinel lymph node
Major	Other Axillary Nodal Procedure	T919	Unspecified operations on sentinel lymph node
Major	Other Axillary Nodal Procedure	T929	Unspecified other operations on lymphatic tissue
Major	Plastics Procedures	B291	Reconstruction of breast using myocutaneous flap of latissimus dorsi muscle
Major	Plastics Procedures	B292	Reconstruction of breast using local flap of skin NEC
Major	Plastics Procedures	B293	Reconstruction of breast using flap of skin of abdomen NEC
Major	Plastics Procedures	B294	Reconstruction of breast using distant flap of skin NEC
Major	Plastics Procedures	B295	Revision of reconstruction of breast
Major	Plastics Procedures	B298	Other specified reconstruction of breast
Major	Plastics Procedures	B299	Unspecified reconstruction of breast
Major	Plastics Procedures	B301	Insertion of prosthesis for breast

Major	Plastics Procedures	B302	Revision of prosthesis for breast
Major	Plastics Procedures	B303	Removal of prosthesis for breast
Major	Plastics Procedures	B308	Other specified prosthesis for breast
Major	Plastics Procedures	B309	Unspecified prosthesis for breast
Major	Plastics Procedures	B311	Reduction mammoplasty
Major	Plastics Procedures	B312	Augmentation mammoplasty
Major	Plastics Procedures	B313	Mastopexy
Major	Plastics Procedures	B314	Revision of mammoplasty
Major	Plastics Procedures	B318	Other specified other plastic operations on breast
Major	Plastics Procedures	B319	Unspecified other plastic operations on breast
Major	Plastics Procedures	B351	Transposition of nipple
Major	Plastics Procedures	B354	Plastic operations on nipple
Major	Plastics Procedures	B356	Eversion of nipple
Major	Plastics Procedures	B358	Other specified operations on nipple
Major	Plastics Procedures	B359	Unspecified operations on nipple
Major	Plastics Procedures	B361	Reconstruction of nipple
Major	Plastics Procedures	B362	Nipple sharing using other tissue
Major	Plastics Procedures	B363	Nipple sharing NEC
Major	Plastics Procedures	B364	Tattooing of nipple
Major	Plastics Procedures	B368	Other specified reconstruction of nipple and areola
Major	Plastics Procedures	B369	Unspecified reconstruction of nipple and areola
Major	Plastics Procedures	B381	Reconstruction of breast using free superior gluteal artery perforator flap
Major	Plastics Procedures	B382	Reconstruction of breast using free inferior gluteal artery perforator flap
Major	Plastics Procedures	B388	Other specified reconstruction of breast using flap of skin of buttock
Major	Plastics Procedures	B389	Unspecified reconstruction of breast using flap of skin of buttock
Major	Plastics Procedures	B391	Reconstruction of breast using free transverse rectus abdominis myocutaneous (TRAM) flap
Major	Plastics Procedures	B392	Reconstruction of breast using pedicled transverse rectus abdominis myocutaneous (TRAM) flap
Major	Plastics Procedures	B393	Reconstruction of breast using free deep inferior epigastric perforator (DIEP)flap
Major	Plastics Procedures	B398	Other specified reconstruction of breast using transverse rectus abdominis myocutaneous (TRAM)
Major	Plastics Procedures	B399	Unspecified reconstruction of breast using transverse rectus abdominis myocutaneous (TRAM)
Major	Total Mastectomy	B271	Total mastectomy and excision of both pectoral muscles and part of chest wall
Major	Total Mastectomy	B273	Patey mastectomy /modified radical mastectomy (plus node excision T852), Total mastectomy and excision of pectoralis minor muscle
Major	Total Mastectomy	B274	Total mastectomy NEC, inc toilet and simple mastectomy, extended simple mastectomy.
Major	Total Mastectomy	B275	Subcutaneous mastectomy
Major	Total Mastectomy	B276	Skin sparing mastectomy
Major	Total Mastectomy	B278	Total excision of breast other specified.
Major	Total Mastectomy	B279	Unspecified, Mastectomy NEC.
Major	Total Mastectomy + Axillary Dissection	B272	Radical mastectomy/total mastectomy and excision of both pectoral muscles NEC.
Minor		B321	Percutaneous biopsy of lesion of breast
Minor		B322	Biopsy of lesion of breast NEC
Minor		B329	Unspecified biopsy of breast
Minor		B331	Drainage of lesion of breast
Minor		B332	Capsulotomy of breast
Minor		B333	Exploration of breast
Minor		B338	Other specified incision of breast
Minor		B339	Unspecified incision of breast
Minor		B345	Exploration of mammillary duct NEC
Minor		B348	Other specified operations on duct of breast
Minor		B349	Unspecified operations on duct of breast
Minor		B355	Biopsy of lesion of nipple

Minor	B371	Aspiration of lesion of breast
Minor	B372	Injection into breast
Minor	B373	Extraction of milk from breast
Minor	B379	Unspecified other operations on breast
Minor	T851	Block dissection of cervical lymph nodes
Minor	T863	Sampling of supraclavicular lymph nodes
Minor	T864	Sampling of internal mammary lymph nodes
Minor	T881	Drainage of lesion of cervical lymph node
Minor	T882	Drainage of lesion of axillary lymph node
Minor	T883	Drainage of lesion of inguinal lymph node
Minor	T889	Unspecified drainage of lesion of lymph node
Minor	T891	Reconstruction of lymphatic duct
Minor	T892	Bypass of obstruction of lymphatic duct
Minor	T893	Ligation of lymphatic duct
Minor	T894	Cannulation of lymphatic duct
Minor	T898	Other specified operations on lymphatic duct
Minor	T899	Unspecified operations on lymphatic duct
Minor	T928	Other specified other operations on lymphatic tissue
