# Supplementary material for: How much do tumor stage and treatment explain socioeconomic inequalities in breast cancer survival? Applying causal mediation analysis to population-based data
Source: Eur J Epidemiol. 2016 May 10;31:603–11. doi: 10.1007/s10654-016-0155-5 (PMC4956701; doi:10.1007/s10654-016-0155-5)
Supplement: Supplementary file 2 — Appendix 2. Potential Collider Bias by Studying Incident Breast Cancer Cases. (PDF 215 kb) [file 10654_2016_155_MOESM2_ESM.pdf]

Appendix 2. Potential Collider Bias by Studying Incident Breast Cancer Cases.

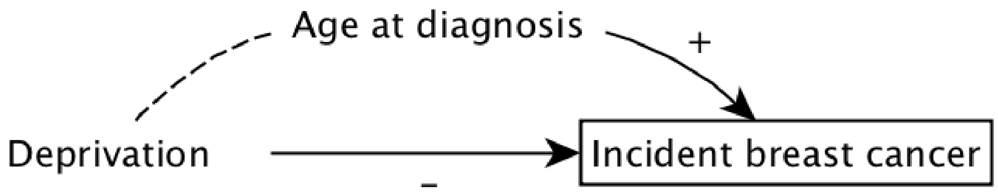

Both socioeconomic position and age may affect breast cancer incidence: conditioning on incident cancer may induce a spurious association between deprivation and age at diagnosis. Because women living in more deprived areas have slightly lower breast cancer incidence rate,[1] and that older women have higher breast cancer incidence, we expect that within those women diagnosed with breast cancer, a small positive association between deprivation and age at diagnosis can be induced. The effect of this induced association on the mediating effects of stage and treatment should be minimal.

In addition, while there is no evidence for under-diagnosis of breast cancer by deprivation [personal communication], over-diagnosis associated with breast cancer screening has been shown to be a minor issue in England.[2] In addition, the vast majority of breast cancers are diagnosed outside of the screening programme.[3] Thus we do not expect to see differences between the more affluent and more deprived breast cancer patients by selection due to cancer screening.

1. National Cancer Intelligence Network. Cancer by deprivation in England: Incidence, 1996-2010, Mortality, 1997-2011. In. United Kingdom: Public Health England 2014; 105.
2. Puliti D, Duffy SW, Miccinesi G et al. Overdiagnosis in mammographic screening for breast cancer in Europe: a literature review. J Med Screen 2012; 19 Suppl 1: 42-56.
3. National Cancer Intelligence Network. Screen-Detected Breast Cancer. In NCIN Data Briefing. 2011; 2.
