# Supplementary material for: How much do tumor stage and treatment explain socioeconomic inequalities in breast cancer survival? Applying causal mediation analysis to population-based data
Source: Eur J Epidemiol. 2016 May 10;31:603–11. doi: 10.1007/s10654-016-0155-5 (PMC4956701; doi:10.1007/s10654-016-0155-5)
Supplement: Supplementary file 3 — Appendix 3. Mathematical Explanations for the Three Mediation Analyses. (PDF 551 kb) [file 10654_2016_155_MOESM3_ESM.pdf]

Appendix 3

Technical details underpinning the three mediation analyses

Analysis one: effect of deprivation on survival status, with stage as mediator

Let $S^*_{most|C}$ be a random draw from the distribution of *Stage* given baseline confounders *C* (i.e. calendar year of diagnosis and region) amongst the most deprived patients, and let $S^*_{least|C}$ be a random draw from the distribution of *Stage* given *C* amongst the least deprived patients. Then, following Vanderweele, Vansteelandt and Robins (Epidemiology, 2014),(1) we define the ***randomized interventional analogue of the natural indirect effect (RIANIE)*** as the log odds ratio of death comparing:

- setting stage to random draws (given baseline confounders) of the distribution of stage amongst the most deprived, to
- setting stage to random draws (given baseline confounders) of the distribution of stage amongst the least deprived,

if, under both scenarios, everyone were set to be most deprived. Written mathematically:

$$\boldsymbol{RIANIE} = log\left(odds\left(Y\left(most, S^*_{most|C}\right)\right)\right) - log\left(odds\left(Y\left(most, S^*_{least|C}\right)\right)\right)$$

where

- *Y* is the survival status, Y=1 for death, 0 for alive
- $Odds(Y) = Pr(Y = 1)/Pr(Y = 0)$
- $Y(most, s)$ is the potential value of *Y* when deprivation is set to *most deprived*, and *Stage* is set to *s*.

The *randomized interventional analogue of natural direct effect (RIANDE)* is the log odds ratio of death, if everyone had their stage set to be a random draw (given confounders) of the distribution of stage amongst the least deprived, comparing setting their deprivation level to most versus least deprived. Written mathematically:

$$\boldsymbol{RIANDE} = log\left(odds\left(Y\left(most, S^*_{least|C}\right)\right)\right) - log\left(odds\left(Y\left(least, S^*_{least|C}\right)\right)\right)$$

The *randomised intervention analogue of the total causal effect (RIATCE)* is the log odds ratio of death comparing:

- setting deprivation to most deprived and stage to a random draw (given baseline confounders) of the distribution of stage amongst the most deprived, versus
- setting deprivation to least deprived and stage to a random draw (given baseline confounders) of the distribution of stage amongst the least deprived.

Written mathematically:

$$\boldsymbol{RIATCE} = log\left(odds\left(Y\left(most, S^*_{most|C}\right)\right)\right) - log\left(odds\left(Y\left(least, S^*_{least|C}\right)\right)\right)$$

This is the sum of the *randomized interventional analogues of the natural direct and indirect effects*.

Similarly, for analysis two: effect of deprivation on survival status, with treatment as mediator

Let $T^*_{most|C}$ be a random draw from the distribution of *Treatment* given baseline confounders *C* (i.e. calendar year of diagnosis and region) amongst the most deprived patients, and let $T^*_{least|C}$ be a random draw from the distribution of *Treatment* given *C* amongst the least deprived patients. Then we have:

$$\boldsymbol{RIANIE} = log\left(odds\left(Y\left(most, T^*_{most|C}\right)\right)\right) - log\left(odds\left(Y\left(most, T^*_{least|C}\right)\right)\right)$$

$$\boldsymbol{RIANDE} = log\left(odds\left(Y\left(most, T^*_{least|C}\right)\right)\right) - log\left(odds\left(Y\left(least, T^*_{least|C}\right)\right)\right)$$

$$\boldsymbol{RIATCE} = log\left(odds\left(Y\left(most, T^*_{most|C}\right)\right)\right) - log\left(odds\left(Y\left(least, T^*_{least|C}\right)\right)\right)$$

where now $Y(most, t)$ is the potential value of *Y* when deprivation is set to *most deprived*, and *Treatment* is set to *t*.

For analysis three: effect of deprivation on treatment, with stage as mediator

$$\boldsymbol{RIANIE} = log\left(odds\left(T\left(most, S^*_{most|C}\right)\right)\right) - log\left(odds\left(T\left(most, S^*_{least|C}\right)\right)\right)$$

$$\boldsymbol{RIANDE} = log\left(odds\left(T\left(most, S^*_{least|C}\right)\right)\right) - log\left(odds\left(T\left(least, S^*_{least|C}\right)\right)\right)$$

$$\boldsymbol{RIATCE} = log\left(odds\left(T\left(most, S^*_{most|C}\right)\right)\right) - log\left(odds\left(T\left(least, S^*_{least|C}\right)\right)\right)$$

where

- *T* is surgical treatment, T=1 for major surgery, 0 for minor/no surgical procedures, and
- $T(most, s)$ is the potential value of *T* when deprivation is set to *most deprived*, and *Stage* is set to s.

The *proportion mediated (PM)* is defined as

$$PM = \frac{RIANIE}{RIATCE}$$

1. VanderWeele TJ, Vansteelandt S, Robins JM. Effect Decomposition in the Presence of an Exposure-Induced Mediator-Outcome Confounder. Epidemiology. 2014;25(2):300-6.
