# Supplementary material for: How much do tumor stage and treatment explain socioeconomic inequalities in breast cancer survival? Applying causal mediation analysis to population-based data
Source: Eur J Epidemiol. 2016 May 10;31:603–11. doi: 10.1007/s10654-016-0155-5 (PMC4956701; doi:10.1007/s10654-016-0155-5)
Supplement: Supplementary file 4 — Appendix 4. Model specifications and STATA codes (PDF 431 kb) [file 10654_2016_155_MOESM4_ESM.pdf]

Appendix 4: STATA Codes and Model Specifications

Defining the outcome variable

```
* Mortality
*mort = dead at `m' months
foreach x in 6 12 36 60 {
      gen mort`x'm = 1 if (dead==1 & time<= `x' / 12)
      replace mort`x'm=0 if mort`x'm==.
}
* mortality at 12m, given 6m survival
gen t_month= time * 12
gen mort12in6m = 1 if (dead==1 & (t_m<=12 & t_m>6))
replace mort12in6m = 0 if (dead==0 & t_m>6) | t_m>12

* mortality at 3 years, given 1 year survival
gen mort3in1y = 1 if (dead==1 & (time<=3 & time>1))
replace mort3in1y = 0 if (dead==0 & time>1) | time>3

* mortality at 5 years, given 3 year survival
gen mort5in3y = 1 if (dead==1 & (time<=5 & time>3))
replace mort5in3y = 0 if (dead==0 & time>3) | time>5

global morttime "6m 12in6m 3in1y 5in3y"

foreach m in $morttime {
preserve
      if "`m'" == "3in1y" | "`m'" == "12in6m" | "`m'" == "5in3y" {
            drop if mort`m'==.
      }
      ***ANALYSIS CODES HERE, SEE BELOW***
restore
}
```

Defining age splines

```
* Age splines
qui sum agediag
global m = r(mean)
gen agediag_ctred = agediag - $m
      * define knots at agediag 50 and 70
      local k0 = 15 - $m
      local k1 = 50 - $m
      local k2 = 70 - $m
      local k3 = 100 - $m
rcsgen agediag_ctred, /*df(2)*/ gen(rcs_age) orthog knots(`k0' `k1'
`k2' `k3')
matrix Rage =r(R)
matrix list Rage
global knotsage `r(knots)'
di $knotsage
```

Analysis one: effect of deprivation on survival status, with stage as mediator

```
gformula mort`m' dep stage rcs_age1 rcs_age2 rcs_age3 ydiag GOR ttt2
dead ftime treatment, mediation ///
equations(stage:i.dep rcs_age1 rcs_age2 rcs_age3 i.GOR ydiag ///
      , mort`m':i.stage i.dep i.dep#i.stage rcs_age1 rcs_age2
      rcs_age3 i.GOR ydiag i.stage#c.rcs_age1 i.stage#c.rcs_age2
      i.stage#c.rcs_age3 ///
      , rcs_age1: i.dep i.GOR ydiag ///
      , rcs_age2: i.dep i.GOR ydiag ///
      , rcs_age3: i.dep i.GOR ydiag) ///
```

```
commands(mort`m':logit, stage:mlogit, rcs_age1:regress,
rcs_age2:regress, rcs_age3:regress) ///
outcome(mort`m') exposure(dep) mediator(stage) post_confs(rcs_age1
rcs_age2 rcs_age3) base_confs(GOR ydiag) ///
                  control(stage:1) baseline(dep:1)  ///
                  oce sam(1000) logOR
```

- We used multinomial logistic regression when modelling stage at diagnosis, and assumed additive effect of deprivation (in 5 categories), age splines, region, and year of diagnosis (continuous) on the log odds scale.
- Splines of age at diagnosis were modelled using linear regression, with additive contribution from deprivation (5 categories), region and year of diagnosis (continuous).
- We used logistic regression when modelling mortality, and included additive effect (on the log odds scale) of stage (4 categories), deprivation (5 categories), age (splines), region, year of diagnosis (continuous), with deprivation and stage interaction, stage and age interaction.

Analysis two: effect of deprivation on survival status, with treatment as mediator

```
gformula mort`m' dep ttt2 stage rcs_age1 rcs_age2 rcs_age3 ydiag GOR
dead ftime treatment, mediation ///
equations(stage:i.dep rcs_age1 rcs_age2 rcs_age3 i.GOR ydiag ///
            , ttt2:i.dep i.stage i.dep#i.stage rcs_age1 rcs_age2
            rcs_age3 i.GOR ydiag i.stage#c.rcs_age1
            i.stage#c.rcs_age2 i.stage#c.rcs_age3 ///
            , mort`m':i.stage i.dep i.ttt2 i.dep#i.stage i.dep#i.ttt2
            i.stage#i.ttt2 i.GOR#i.ttt2 i.stage#c.rcs_age1
            i.stage#c.rcs_age2 i.stage#c.rcs_age3 i.ttt2#c.rcs_age1
            i.ttt2#c.rcs_age2 i.ttt2#c.rcs_age3 rcs_age1 rcs_age2
            rcs_age3 i.GOR ydiag ///
            , rcs_age1: i.dep i.GOR ydiag ///
            , rcs_age2: i.dep i.GOR ydiag ///
            , rcs_age3: i.dep i.GOR ydiag) ///
commands(mort`m':logit, stage:mlogit, ttt2:logit, rcs_age1:regress,
rcs_age2:regress, rcs_age3:regress) ///
outcome(mort`m') exposure(dep) mediator(ttt2) post_confs(stage
rcs_age1 rcs_age2 rcs_age3) base_confs(GOR ydiag) ///
                  control(ttt2:1) baseline(dep:1) ///
                  oce sam(1000) logOR
```

- We used multinomial logistic regression when modelling stage at diagnosis, and assumed additive effect of deprivation (in 5 categories), age splines, region, and year of diagnosis (continuous) on the log odds scale.
- Splines of age at diagnosis were modelled using linear regression, with additive contribution from deprivation (5 categories), region and year of diagnosis (continuous).
- We have modelled treatment (binary) using logistic regression. We included additive effect (on the log odds scale) of stage (4 categories), deprivation (5 categories), age (splines), region, year of diagnosis (continuous), with deprivation and stage interaction, stage and age interaction.
- We used logistic regression when modelling mortality, and included additive effect (on the log odds scale) of stage (4 categories), deprivation (5 categories), treatment (binary), age (splines), region, year of diagnosis (continuous), with deprivation and stage interaction, deprivation and treatment interaction, stage

and treatment interaction, region and treatment interaction, stage and age interaction, and treatment and age interaction.

<u>Analysis three: effect of deprivation on treatment, with stage as mediator</u>

```
gformula dep ttt2 stage rcs_age1 rcs_age2 rcs_age3 ydiag GOR dead
ftime treatment, mediation ///
equations(stage:i.dep rcs_age1 rcs_age2 rcs_age3 i.GOR ydiag ///
            , ttt2:i.stage i.dep i.dep#i.stage rcs_age1 rcs_age2
            rcs_age3 i.GOR ydiag i.stage#c.rcs_age1
            i.stage#c.rcs_age2 i.stage#c.rcs_age3 ///
            , rcs_age1: i.dep i.GOR ydiag ///
            , rcs_age2: i.dep i.GOR ydiag ///
            , rcs_age3: i.dep i.GOR ydiag) ///
commands(stage:mlogit, ttt2:logit, rcs_age1:regress,
rcs_age2:regress, rcs_age3:regress) ///
outcome(ttt2) exposure(dep) mediator(stage) post_confs(rcs_age1
rcs_age2 rcs_age3) ///
      control(stage:1) baseline(dep:1) base_confs(GOR ydiag) ///
      oce sam(1000) logOR
```

- We used multinomial logistic regression when modelling stage at diagnosis, and assumed additive effect of deprivation (in 5 categories), age splines, region, and year of diagnosis (continuous) on the log odds scale.
- Splines of age at diagnosis were modelled using linear regression, with additive contribution from deprivation (5 categories), region and year of diagnosis (continuous).
- We have modelled treatment (binary) using logistic regression. We included additive effect (on the log odds scale) of stage (4 categories), deprivation (5 categories), age (splines), region, year of diagnosis (continuous), with deprivation and stage interaction, stage and age interaction.
