# Supplementary material for: How much do tumor stage and treatment explain socioeconomic inequalities in breast cancer survival? Applying causal mediation analysis to population-based data
Source: Eur J Epidemiol. 2016 May 10;31:603–11. doi: 10.1007/s10654-016-0155-5 (PMC4956701; doi:10.1007/s10654-016-0155-5)
Supplement: Supplementary file 5 — Appendix 5. One-year and Five-year Net Survival for Women Diagnosed with Breast Cancer, Yorkshire and North East (England), 2000-07. (PDF 237 kb) [file 10654_2016_155_MOESM5_ESM.pdf]

Appendix 5: One-year and five-year net survival for women diagnosed with breast cancer, Yorkshire and North East (England), 2000-07

		STAGE ALL			STAGE I			STAGE II			STAGE III			STAGE IV			MISSING		
**Year**	**Deprivation**	**NS**	**lci**	**uci**	**NS**	**lci**	**uci**	**NS**	**lci**	**uci**	**NS**	**lci**	**uci**	**NS**	**lci**	**uci**	**NS**	**lci**	**uci**
One year after diagnosis	Least deprived	0.97	(0.97	- 0.98)	1.00	(1.00	- 1.00)	0.99	(0.99	- 1.00)	0.95	(0.92	- 0.97)	0.55	(0.48	- 0.62)	0.90	(0.86	- 0.94)
	2	0.96	(0.96	- 0.97)	1.00	(0.99	- 1.00)	0.99	(0.99	- 1.00)	0.89	(0.86	- 0.92)	0.55	(0.49	- 0.61)	0.85	(0.81	- 0.88)
	3	0.95	(0.95	- 0.96)	0.99	(0.99	- 1.00)	0.99	(0.98	- 1.00)	0.91	(0.88	- 0.94)	0.54	(0.48	- 0.60)	0.84	(0.81	- 0.88)
	4	0.94	(0.94	- 0.95)	1.00	(0.99	- 1.00)	0.98	(0.97	- 0.99)	0.91	(0.88	- 0.94)	0.51	(0.46	- 0.57)	0.82	(0.78	- 0.85)
	Most deprived	0.94	(0.93	- 0.94)	1.00	(0.99	- 1.00)	0.98	(0.98	- 0.99)	0.92	(0.89	- 0.95)	0.44	(0.40	- 0.49)	0.80	(0.77	- 0.84)
Five years after diagnosis	Least deprived	0.86	(0.84	- 0.87)	0.96	(0.94	- 0.98)	0.86	(0.84	- 0.89)	0.66	(0.58	- 0.73)	0.12	(0.06	- 0.17)	0.75	(0.65	- 0.85)
	2	0.83	(0.82	- 0.85)	0.96	(0.94	- 0.98)	0.84	(0.82	- 0.87)	0.60	(0.53	- 0.67)	0.14	(0.08	- 0.19)	0.62	(0.55	- 0.70)
	3	0.82	(0.80	- 0.83)	0.94	(0.92	- 0.96)	0.86	(0.83	- 0.88)	0.61	(0.54	- 0.69)	0.14	(0.08	- 0.20)	0.57	(0.49	- 0.64)
	4	0.80	(0.78	- 0.82)	0.96	(0.94	- 0.98)	0.84	(0.82	- 0.87)	0.52	(0.45	- 0.59)	0.14	(0.09	- 0.19)	0.56	(0.48	- 0.64)
	Most deprived	0.76	(0.74	- 0.78)	0.95	(0.92	- 0.97)	0.80	(0.77	- 0.82)	0.53	(0.46	- 0.60)	0.11	(0.07	- 0.15)	0.49	(0.41	- 0.56)
