# Supplementary material for: How much do tumor stage and treatment explain socioeconomic inequalities in breast cancer survival? Applying causal mediation analysis to population-based data
Source: Eur J Epidemiol. 2016 May 10;31:603–11. doi: 10.1007/s10654-016-0155-5 (PMC4956701; doi:10.1007/s10654-016-0155-5)
Supplement: Supplementary file 6 — Appendix 6: Effects of Deprivation on Mortality, Mediated via Stage at Diagnosis. (PDF 325 kb) [file 10654_2016_155_MOESM6_ESM.pdf]

Appendix 6: Effects of deprivation on mortality, mediated via stage at diagnosis

	Deprivation	Effect: Total OR	Total LCI	Total UCI	via stage OR	via stage LCI	via stage UCI	Proportion mediated: via stage§ PM	via stage§ LCI	via stage§ UCI	from sensitivity analysis* PM	from sensitivity analysis* LCI	from sensitivity analysis* UCI
**At 6 months**	*Least deprived*	*Baseline*											
	2	1.42	1.14	1.76	0.82	0.73	0.91	-0.59	-2.14	0.96	-0.02	-2.27	2.23
	3	1.98	1.60	2.45	0.97	0.88	1.08	-0.04	-0.23	0.15	-0.05	-0.19	0.09
	4	2.22	1.77	2.77	0.92	0.84	1.01	-0.10	-0.23	0.03	0.01	-0.12	0.15
	Most deprived	2.77	2.17	3.53	1.43	1.27	1.62	0.35	0.23	0.48	0.25	0.05	0.46
**At 1 year** conditioning on 6-month survival	*Least deprived*	*Baseline*											
	2	1.29	0.98	1.71	0.91	0.81	1.02	-0.38	-13.19	12.44	-0.84	-7.02	5.35
	3	1.47	1.12	1.93	1.02	0.92	1.13	0.05	-1.24	1.33	0.00	-1.98	1.98
	4	1.75	1.35	2.27	1.10	1.00	1.22	0.17	-0.08	0.43	-0.08	-1.14	0.98
	Most deprived	1.93	1.49	2.50	1.21	1.08	1.36	0.30	0.05	0.54	0.42	-1.33	2.18
**At 3 years** conditioning on 1-year survival	*Least deprived*	*Baseline*											
	2	1.38	1.19	1.60	0.93	0.87	0.99	-0.24	-0.54	0.06	-0.14	-0.53	0.24
	3	1.40	1.21	1.62	0.96	0.90	1.02	-0.12	-1.10	0.86	0.00	-0.83	0.83
	4	1.70	1.48	1.96	0.98	0.92	1.04	-0.04	-0.16	0.09	-0.03	-0.16	0.10
	Most deprived	1.98	1.73	2.26	1.09	1.03	1.15	0.12	0.04	0.21	0.18	0.05	0.30
**At 5 years** conditioning on 3-year survival	*Least deprived*	*Baseline*											
	2	1.10	0.90	1.33	1.01	0.93	1.09	0.11	-8.74	8.96	0.08	-9.96	10.12
	3	1.26	1.04	1.52	1.03	0.95	1.11	0.13	-2.12	2.39	0.08	-3.70	3.85
	4	1.33	1.10	1.61	1.02	0.94	1.10	0.06	-3.07	3.19	0.01	-4.13	4.16
	Most deprived	1.67	1.39	2.00	1.08	1.00	1.16	0.14	-0.03	0.31	0.22	-0.70	1.13

§ The null hypothesis is 0, i.e. no effect is mediated via the mediator(s). A PM of 1 means that all of the total effect is mediated via the mediator(s)

* Sensitivity analysis: both age and stage at diagnosis are considered as mediators together
