# Supplementary material for: How much do tumor stage and treatment explain socioeconomic inequalities in breast cancer survival? Applying causal mediation analysis to population-based data
Source: Eur J Epidemiol. 2016 May 10;31:603–11. doi: 10.1007/s10654-016-0155-5 (PMC4956701; doi:10.1007/s10654-016-0155-5)
Supplement: Supplementary file 7 — Appendix 7: Effects of Deprivation on Mortality, Mediated via Surgical Treatment. (PDF 324 kb) [file 10654_2016_155_MOESM7_ESM.pdf]

Appendix 7: Effects of deprivation on mortality, mediated via surgical treatment

		**Effect:**						**Proportion mediated:**					
	**Deprivation**	**Total**			**via treatment**			**via treatment§**			**from sensitivity analysis***		
		OR	LCI	UCI	OR	LCI	UCI	PM	LCI	UCI	PM	LCI	UCI
**At 6 months**	*Least deprived*	*Baseline*											
	2	1.87	1.01	3.47	1.07	0.96	1.19	0.11	-0.84	1.06	-0.17	-2.39	2.05
	3	3.00	1.58	5.70	1.08	0.98	1.19	0.07	-0.31	0.45	0.06	-0.09	0.22
	4	2.75	1.46	5.17	1.02	0.92	1.13	0.02	-0.16	0.19	0.09	-0.06	0.24
	Most deprived	2.99	1.55	5.76	0.94	0.85	1.03	-0.06	-0.26	0.14	-0.10	-0.26	0.05
**At 1 year** conditioning on 6 month survival	*Least deprived*	*Baseline*											
	2	1.18	0.61	2.32	1.06	0.91	1.23	0.33	-3.81	4.48	0.06	-2.23	2.36
	3	1.26	0.65	2.43	0.90	0.79	1.03	-0.44	-1.49	0.62	-0.02	-4.94	4.91
	4	1.23	0.64	2.35	0.87	0.76	1.00	-0.68	-5.44	4.08	0.17	-0.45	0.79
	Most deprived	1.74	0.90	3.37	0.99	0.86	1.13	-0.02	-1.72	1.67	-0.22	-1.01	0.57
**At 3 years** conditioning on 1 year survival	*Least deprived*	*Baseline*											
	2	1.51	1.09	2.08	1.04	0.96	1.12	0.09	-1.32	1.50	0.12	-1.66	1.90
	3	1.38	0.98	1.93	0.98	0.91	1.06	-0.07	-0.77	0.63	0.00	-0.52	0.51
	4	1.74	1.26	2.39	1.00	0.93	1.07	0.00	-0.12	0.12	0.00	-0.13	0.12
	Most deprived	2.08	1.52	2.86	0.98	0.91	1.06	-0.02	-0.14	0.09	-0.08	-0.22	0.07
**At 5 years** conditioning on 1 year survival	*Least deprived*	*Baseline*											
	2	1.12	0.78	1.61	1.02	0.93	1.13	0.22	-9.91	10.35	4.92	-1.03	10.87
	3	1.37	0.98	1.93	1.01	0.93	1.10	0.04	-5.01	5.08	0.18	-10.71	11.07
	4	1.21	0.85	1.71	0.99	0.90	1.08	-0.07	-6.83	6.68	0.29	-4.73	5.30
	Most deprived	1.52	1.08	2.14	0.95	0.87	1.03	-0.13	-5.17	4.92	-0.06	-0.83	0.72

§ The null hypothesis is 0, i.e. no effect is mediated via the mediator(s). A PM of 1 means that all of the total effect is mediated via the mediator(s)

* Sensitivity analysis: three categories of treatment, instead of two, is used as the mediator
