# Supplementary material for: How much do tumor stage and treatment explain socioeconomic inequalities in breast cancer survival? Applying causal mediation analysis to population-based data
Source: Eur J Epidemiol. 2016 May 10;31:603–11. doi: 10.1007/s10654-016-0155-5 (PMC4956701; doi:10.1007/s10654-016-0155-5)
Supplement: Supplementary file 8 — Appendix 8: Effects of Deprivation on Receiving Major Surgical Treatment, Mediated via Stage at Diagnosis. (PDF 308 kb) [file 10654_2016_155_MOESM8_ESM.pdf]

Appendix 8: Effects of deprivation on treatment, mediated via stage

**Deprivation**	**Total effect**			**Effect via stage**			**Proportion mediated via stage (PM)[§]**			**PM from sensitivity analysis***		
	OR	LCI	UCI	OR	LCI	UCI	PM	LCI	UCI	PM	LCI	UCI
Least deprived	*Baseline*											
2	1.05	(0.97 -	1.14)	1.07	(1.04 -	1.11)	1.35	(-29.69 -	32.40)	4.96	(-49.69 -	59.62)
3	0.94	(0.86 -	1.02)	0.98	(0.95 -	1.02)	0.24	(-15.44 -	15.93)	1.56	(-28.00 -	31.12)
4	0.97	(0.89 -	1.05)	1.03	(0.99 -	1.07)	-0.83	(-20.35 -	18.68)	-1.28	(-15.02 -	12.47)
Most deprived	0.94	(0.87 -	1.02)	0.91	(0.88 -	0.94)	1.49	(-60.46 -	63.44)	-0.66	(-7.51 -	6.19)

§ The null hypothesis is 0, i.e. no effect is mediated via the mediator(s). A PM of 1 means that all of the total effect is mediated via the mediator(s)

* Sensitivity analysis: both age and stage at diagnosis are considered as mediators together
